# LINC81507 act as a competing endogenous RNA of miR-199b-5p to facilitate NSCLC proliferation and metastasis via regulating the CAV1/STAT3 pathway

**DOI:** 10.1038/s41419-019-1740-9

**Published:** 2019-07-11

**Authors:** Wei Peng, Dan He, Bin Shan, Jun Wang, Wenwen Shi, Wenyuan Zhao, Zhenzi Peng, Qingxi Luo, Minghao Duan, Bin Li, Yuanda Cheng, Yeping Dong, Faqing Tang, Chunfang Zhang, Chaojun Duan

**Affiliations:** 1 Department of Oncology, Xiangya Hospital, Central South University, Changsha, 410008 PR China; 2 Hunan Cancer Hospital, The Affiliated Tumor Hospital of Xiangya Medical College, Central South University, Changsha, 410008 PR China; 3 College of Medicine, Washington State University Spokane, Spokane, WA 99201 USA; 4 National Clinical Research Center for Geriatric Disorders, Xiangya Hospital, Central South University, Changsha, 410008 PR China; 5 Department of Thoracic Surgery, Xiangya Hospital, Central South University, Changsha, 410008 PR China

**Keywords:** Non-small-cell lung cancer, Long non-coding RNAs

## Abstract

Lung cancer is the leading cause of cancer-related mortality worldwide. Recently, accumulating data indicate that long noncoding RNAs (LncRNAs) function as novel crucial regulators of diverse biological processes, including proliferation and metastasis, in tumorigenesis. Lnc NONHSAT081507.1 (LINC81507) is associated with lung adenocarcinoma. However, its pathological role in non-small cell lung cancer (NSCLC) remains unknown. In our study we investigated the role of LINC81507 in NSCLC. The expression of LINC81507 was analyzed in 105 paired NSCLC tumor specimens and paired adjacent non-tumorous tissues from NSCLC patients by real-time quantitative PCR (RT-qPCR). Gain- and loss-of-function experiments were conducted to investigate the functions of LINC81507, miR-199b-5p and CAV1. Reduced expression of LINC81507 resulted in cell growth, proliferation, migration and epithelial–mesenchymal transition (EMT) in NSCLC cells, whereas ectopic overexpression of LINC81507 resulted in the opposite effects both in vitro and in vivo. Nuclear and Cytoplasmic fractionation assays showed LINC81507 mainly resided in cytoplasm. Bioinformatics analysis and dual-luciferase assays revealed that miR-199b-5p was a direct target of LINC81507 through binding Ago2. Mechanistic analysis demonstrated that miR-199b-5p specifically targeted the Caveolin1 (CAV1) gene, and LINC81507 inactivated the STAT3 pathway in a CAV1-dependent manner. Taken together, LINC81507 is decreased in NSCLC and functions as a sponge to miR-199b-5p to regulate CAV1/STAT3 pathway, which suggests that LINC81507 serve as a tumor suppressor and potential therapeutic target and biomarker for metastasis and prognosis in NSCLC.

## Introduction

According to the Cancer Statistics released by American Cancer Society in 2018, lung cancer is not only the most frequently diagnosed cancer but also the leading cause of cancer death worldwide^1^. Non-small cell lung carcinoma (NSCLC) consist of almost 80% of lung cancer. NSCLC is the leading cause of cancer-related deaths in United States and in China^1–3^. Lack of obvious symptoms delays early diagnosis for lung cancer patients. Although the surgical comprehensive treatment has achieved great progress, 5-year survival rate is still <15%^4^. Most NSCLC patients were diagnosed at an advanced stage. Accordingly, the molecular mechanisms’ study of non-small cell lung cancer’ proliferation and metastasis can provide guidance for development of diagnosis, prognostic and therapeutic strategies. Emerging evidence shows that although long noncoding RNAs (LncRNAs) lack the protein coding potential, they can control transcription, translation and protein function through various mechanisms^5^. More and more attentions had been focused on LncRNAs, especially in cancer.

LncRNAs are a diverse class of RNAs whose length is longer than 200 nucleotides^6^. Recently, Iyer and his colleges showed that there are about 60,000 noncoding RNAs in the human genome while almost 42,000 are lncRNAs^7^. However, the total number is still climbing. About 80% of lncRNAs are not fully elaborated. LncRNAs could engage in various biological processes fuse in every branch of life. LncRNAs can act as scaffolds, decoys and signal transducers via cis and trans manners^8–10^. LncRNAs have the potential in NSCLC diagnosis and prognosis^11^. Moreover, lncRNA-P53RRA can promote ferroptosis and apoptosis in cancer via sequestration of p53^12^. Several lines of evidence emphasizes that lncRNAs function as competitive endogenous RNA (ceRNA) for miRNAs in NSCLC^13,14^. OIP5-AS1 can promote lung cancer cells’ proliferation by targeting miR-378a-3p^15^. LINC00339 sponges miR-145 and interferes FOXM1 to facilitate NSCLC tumorigenesis^16^. MALAT1 activates Akt/mTOR signaling via targeting miR-206 to accelerate NSCLC cells migration and invasion^17^.

Lnc NONHSAT081507.1 (LINC81507) was first identified by our team using Agilent Human LncRNA Array^18^. We found that LINC81507 expression was lower in NSCLC including adenocarcinoma and squamous cell carcinoma when compared with adjacent normal tissues, especially in the tumors with lymph node metastasis. We also found that LINC81507 acts as a ceRNA for miR-199b-5p through direct binding and interferes miR-199b-5p-mediated regulation of CAV1, reduces migration and invasion. In addition, the expression of LINC81507 were positively correlated with 3-year survival. Thus, LINC81507 is an independent predictor for the overall survival in NSCLC. In summary, our findings demonstrate tumor suppressive functions of LINC81507 in NSCLC progression and LINC81507 can serve as a therapeutic target and potential biomarker for diagnosis and prognosis NSCLC.

## Methods

### Patient and specimens

The total 105 cancer tissues and paired adjacent non-tumor tissues (ANLTs, which obtained at least 5 cm away from the tumor edge) were obtained from patients with NSCLC during operation at the Department of Thoracic Surgery, Xiangya Hospital of Central South University (Changsha, China) from January 2012 to July 2016. Clinicopathological TNM staging was judged according to the 8th lung cancer TNM classification criteria. The clinicopathological characteristics of 105 patients were summarized in Table 1. All the fresh specimens were rapidly frozen in liquid nitrogen, and then stored in −80 °C refrigerator.

**Table 1:** Correlation analysis of clinicopathological features between LINC 81507 and NSCLC patients

Characteristics	Number	LINC81507 expression		*P* value
		Low (*n*)	High (*n*)	
*Age (years)*				
≤60	40	30	10	0.965
>60	65	49	16	
*Pathological type*				
Adenocarcinoma	83	60	23	0.174
Squamous cell carcinoma	22	19	3	
*Gender*				
Male	64	45	19	0.144
Female	41	34	7	
*Tumor invasion*				
T1	12	11	1	0.003
T2	70	56	14	
T3	13	9	4	
T4	10	3	7	
*Lymph node metastasis*				
N0	69	60	9	<0.01
N1	22	13	9	
N2 + N3	14	6	8	
*Distant metastasis*				
M0	90	72	18	0.006
M1	15	7	8	
*TNM stage*				
I	47	34	13	<0.05
II	40	36	4	
III + IV	18	9	9	

### Cell line

Five human lung cancer cell lines (PC-9, H1299, A549, H1975 and Calu-1) were purchased from Chinese Academy of Sciences Cell Bank (Shanghai, China). SK-MES-1 were purchased from Shanghai Institutes for Biological Sciences, CAS. PC-9, H1299, A549, H1975, Calu-1 and human bronchial epithelium cells (HBE) were cultured in RPMI 1640 medium containing 10% fetal bovine serum (FBS) and 1% penicillin-streptomycin (P/S) at 37 °C and 5% CO_2_, SK-MES-1 was maintained in MEM medium containing 10% FBS and P/S. All cell lines were passaged <10 times after the initial revival from frozen stocks. All cell lines were authenticated prior to use by short tandem repeat profiling.

### RNA extraction and real-time quantitative polymerase chain reaction

Total RNA was isolated from cells and frozen tissue specimens using TRIzol reagent (Invitrogen, Carlsbad, CA). cDNA was generated using GoScript Reverse Transcription System (Promega, USA). The RNA levels were quantified using SYBR Green qPCR Mix (GeneCopoeia, USA). 36B4, U6 and Actin were used to normalize LncRNAs, miRNAs and mRNAs. The primers were listed in Table 2.

**Table 2:** Primer sequence

Lnc NONHSAT081507.1	
F	TACTGTTTCCAAACTGGACACTGGAGAATATTCC
R	TTTTTTAAGTTAGTTTATACAAATGCCTATGAAATCAAATTAGTGACCA
miRNA 199b-5p	CGCGCGACCCAGTGTTTAGACTATCTGTTC
miRNA 30e-5p	GCCGCGTGTAAACATCCTTGACTGGAAG
miRNA 106a	GCGAAAAGTGCTTACAGTGCAGGTAG
CAV1	
F	CATCCCGATGGCACTCATCTG
R	TGCACTGAATCTCAATCAATCAGGAAG
CAV1-shRNA	
1	GACGTGGTCAAGATTGACTTTTCAAGAGAAAGTCAATCTTGACCACGTCTTTTTT
2	GCCACCTTCACTGTGACGAAATTTCAAGAGAATTTCGTCACAGTGAAGGTGGTTTTTT
3	GACCCACTCTTTGAAGCTGTTTTCAAGAGAAACAGCTTCAAAGAGTGGGTCTTTTTT
36B4	
F	CAACCCAGCTCTGGAGAAAC
R	GTGAGGTCCTCCTTGGTGAA
U6	
F	CGAACGATACAGAGAAGATTAGC
R	TGGAACGCTTCACGAATTTGCG
Actin	
F	CCTGTACGCCAACACAGTGC
R	ATACTCCTGCTTGCTGATCC
GAPDH (nuclear)	
F	GGGAGCCAAAAGGGTCAT
R	GAGTCCTTCCACGATACCAA
U6 (cytoplamic)	
F	CTCGCTTCGGCAGCACA
R	AACGCTTCACGAATTTGCGT
Probe of FISH	
1 TTCCA GTAAG TATAC TTTCC TACAA TGCAT GTCTT TCTCC	
2 AGGTT TAATT GGCTC ACGGT TCTGC AAGCT GTACA ATCCT	
3 ACTCT ATCAA CATCC CACTT CACAC TTCAT ACCTA CTTGT	

### Fluorescent in situ hybridization (FISH) assays

In situ detection of lncRNA was performed in lung cancer tissues using BOSTER kit. Fluorescence-labeled probes for LINC81507 were presented in Table 2.

### Nuclear and cytoplasmic fractionation

Nuclear and cytoplasmic fractionation using PARIS™ Kit (Invitrogen™, AM1921) were performed in A549 and Calu-1 cells. The RNA expression was measured using RT-qPCR.

### Cell transfection, lentivirus production, and transduction

The SMARTer™ RACE cDNA kit (Takara) was used to obtain the full sequence of LINC81507. The pcDNA3.1 vector for LINC81507 overexpression and negative control were constructed by Shanghai Genechem. Cells were harvested for analysis at 48 h after transfection. After lentivirus transduction, PC-9, A549, and Calu-1 cells were treated with 1 mg/ml puromycin to establish stable cell lines. The hsa-miR-199b-5p mimic, hsa-miR-199b-5p inhibitor, and negative control (NC) oligonucleotides were purchased from Ruibio (Guangzhou, China). The pcDNA3.1-CAV1, pcDNA3.1 vectors, pLent-CAV1-shRNA, and pLent vectors were purchased from Vigene Biosciencces (USA). The cell transfections were performed using lipofectamine 3000 (Invitrogen).

### Cell counting kit-8 assays

Cell proliferation was monitored using cell counting kit-8 (CCK8) (Beyotime Biotechnology). A549, PC-9, and Calu-1 cells (3 × 10^3^/well) were seeded in 96-well plates. Cell proliferation was documented on 24, 48, 72, and 96 h. The experiments were repeated three times.

### Colony formation assays

After transfection, A549, PC-9 (2 × 10^2^/well) and Calu-1 cells (7 × 10^2^/well) were cultured in 6-well plates for 14 days. Paraformaldehyde fix solution (4% PFS) and 0.1% crystal violet (Beyotime Biotechnology) were added into 6-well plates. Visible cell colonies were then counted.

### 5-ethylnyl-2ʹ-deoxyuridine (EdU) incorporation assay

EdU assay was performed in Cell-Light™ EdU Apollo 488 In Vitro Kit (Ruibio, China). A549, PC9, and Calu-1 cells (1 × 10^4^) were incubated in 96-well plates after plasmid transfection. Hoechst 33,342 was used to identify nucleic acid in these three cell lines under a fluorescence microscope.

### Migration and invasion assays

The cells (4 × 10^4^/100 µl) in serum-free media were seeded into the upper chambers that were covered with (invasion) or without (migration) Matrigel (BD, USA). The lower chambers contained 600 µl 1640 medium containing antibiotics and 10% FBS. After 36 h, the cells that had migrated or invaded through the upper chamber were dyed with 4% PFS and 0.1% crystal violet. Then, an inverted microscope (Canon, Japan) was used to count and capture these images.

### Gelatin zymography

Gelatin zymography was a classical method to detect gelatinases in the supernatants or cell lysates^19^. The supernatants and cell lysates of A549 and Calu-1 cells (which contained 10 µl supernatants/proteins of cell lysate and 10 µl native gel loading buffer) were loaded on a gelatin (G-8061, Solarbio) containing 10% SDS polyacrylamide gels followed by electrophoresis for 140 V–100 min at 4 ℃. Triton X-100 was used to wash the gels to renature MMP proteins, followed by incubation for 36 h at 37 °C. Gels were stained with Coomassie blue staining for 90 min and then tarnished for 60 min until bands visible.

### Bioinformatics methods

Starbase, NONCODE 2.0, TANRIC and LNCipedia were used to predict LINC81507 potential target microRNA. Targetscan, miRanda and mirBase were used to predict potential target gene, combined with the literature and through the test screening, miR-199b-5p and CAV1 were selected for further studies.

### Luciferase reporter assay

293T cells were seeded in a 24-well plates. Twenty-four hours after co-transfected with miR-199b-5p mimics/inhibitors and the corresponding luciferase reporter vectors (LINC81507 and CAV1 as indicated), dual luciferase reporter assay system (Promega, Madison, WI, USA) were applied.

### RNA-binding protein immunoprecipitation assay

Magna RNA immunoprecipitation (RIP) kit (Millipore, USA) was conducted to finish RIP assay. A549 cell lysate was incubated in RIP buffer that contained magnetic beads and was conjugated with human anti-Ago2 antibody, input or normal rabbit IgG were included as negative control. Proteinase K was used in purifying the immunoprecipitated RNAs. RT-qPCR were performed to detect the binding of target LINC81507 and miR-199b-5p.

### Xenografted tumor model

BALB/c-nu nude male mices (age of 3–4 weeks, 16–18 g) were obtained from the Hunan Slack King of Laboratory Animal Co., Ltd (Changsha, China). For the in vivo tumor proliferation assay, 1 × 10^7^ A549 cells transfected with LINC81507-overexpression (LINC81507-OE) or LINC81507-control (LINC81507-ctrl) were injected subcutaneously into nude mice (6 per group). The tumor volume was monitored and recorded once a week. Tumor volume was calculated as follows: volume = length × width^2^/2. 25 days later, the mices were sacrificed. For in vivo metastasis assays, 1 × 10^6^ A549^LINC81507-OE^ cells and A549^LINC81507-ctrl^ cells were injected intravenously into the tail vein of mice. Each animal was injected with 2 nmol MMP Sense 750 FAST (PerkinElmer, USA) 60 days later. At 12 h after injection, mices were imaged by Fluorescence Molecular Tomography system (PerkinElmer, USA) and analyzed using corresponding software (True Quant, PerkinElmer, USA). All mices were sacrificed, and metastatic nodules were calculated. Then, lung tissues were subjected to immunohistochemical analysis.

### Western blot assay

The total proteins were obtained from cells and then quantified using BCA method. These protein samples were separated through sodium dodecyl sulfate-polyacrylamide gel electrophoresis (SDS-PAGE), and then transferred to a methanol activated PVDF membrane. After blocking with skim milk at the room temperature for 1 h, the membrane was incubated in primary antibodies in a proper dilution overnight at 4 °C. After that, the membranes were incubated with rabbit or mouse secondary antibodies for 1 h at the room temperature and then visualized using chemiluminescence reagents.

### Immunohistochemistry staining (IHC)

IHC analysis was performed on a 60 paraffin-embedded NSCLC tissue microarrays, using anti-CAV1 (1:500, ab32577, Abcam). Then, we performed HE and IHC in the lungs, using the following primary antibodies, anti-E-cadherin (1:500, ab76055, Abcam), anti-N-cadherin (1:200, ab18203, Abcam), anti-β-catenin (1:200, ab32572, Abcam), anti-Vimentin (1:200, ab92547, Abcam).

### Statistical analysis

All statistical analyses were performed using SPSS 17.0 software and Graph Pad Prism. These experiments were performed independently at least three times. Student's *t*-test or one-way ANOVA was used to analyze the mean between different groups. LINC81507 expression and clinicopathological variables was calculated by the χ test or Fisher’s exact test. Survival curves were established using the Kaplan–Meier method and compared by the log-rank test. *P* < 0.05 was considered statistically significant.

## Results

### Lower expression of LINC81507 was associated with advanced TNM staging and metastasis of NSCLC

**Fig. 1:**
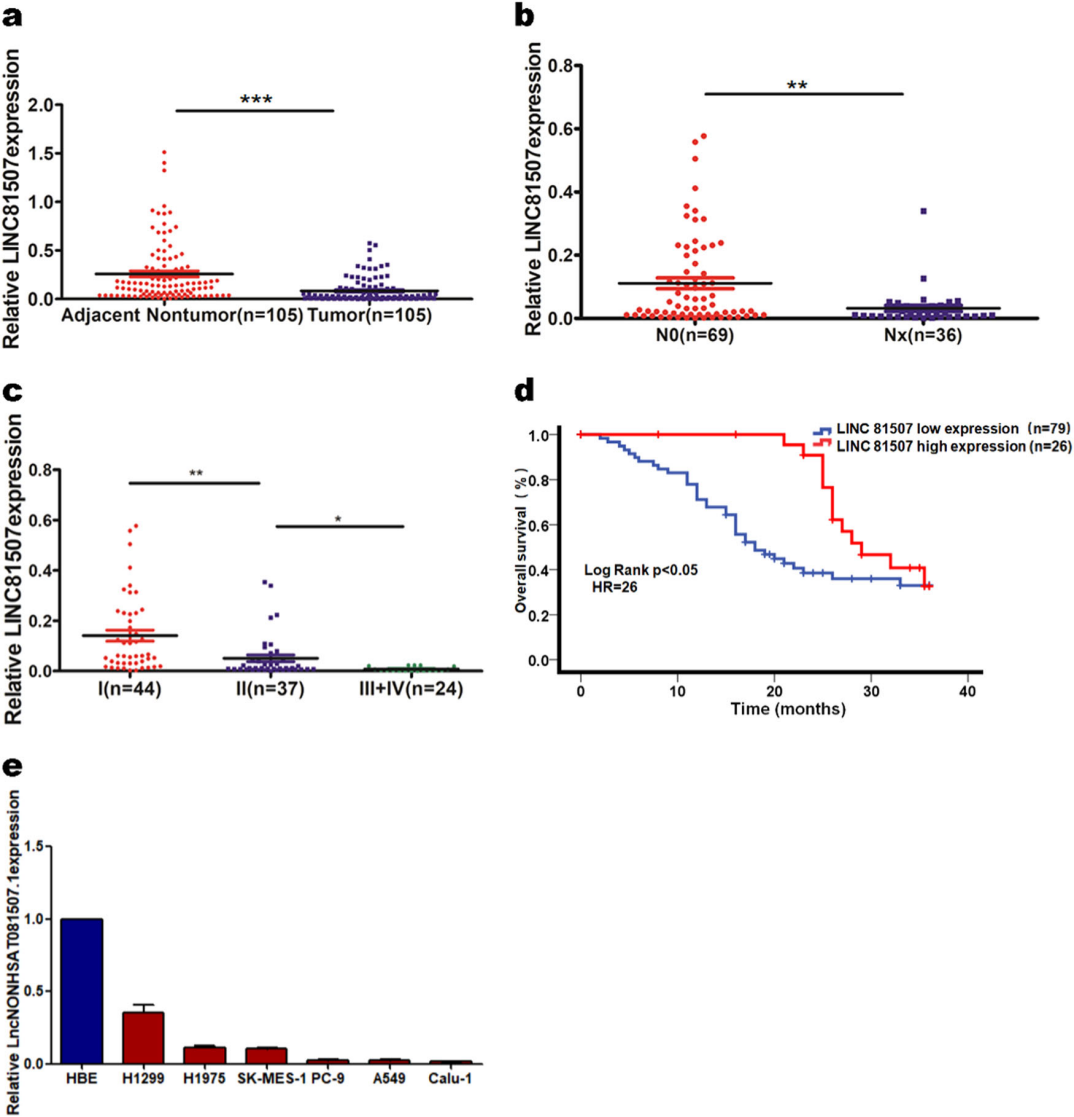
LncRNA-LINC81507 expression profile and survival curve in NSCLC. **a** LINC81507 expression were evaluated using RT-qPCR in 105 pairs of NSCLC tissues and the matched adjacent normal tissues. **b** LINC81507 expression in NSCLC patients with/without node metastasis. _1+2+3_ Nx means the patients who have node metastasis. **c** LINC81507 expression in NSCLC patients whose stages were I/II/III+IV. **d** Kaplan–Meier survival curve indicated the high LINC81507 expression is correlated with well survival rates. **e** LINC81507 expression in NSCLC cell lines and normal human bronchial epithelial cell line. Data were represented as the mean ± SEM of three independent experiments. *P < 0.05, **P < 0.01, ***P < 0.001

We previously reported that LINC81507 was differentially expressed in ADC^18^. According to NONCODE 2.0 and LNCipedia database annotation, LINC81507 is located on chr21: 27074489–27082491 and transcribed into a 946 nt long lncRNA^18^ (additional file. 2a). In this study, we finished the RT-qPCR in 105 NSCLC tumor tissues and paired adjacent normal lung tissues to measure the expression of LINC81507. As shown in Fig. 1a, we compared LINC81507 expression in NSCLC tissues with ANLTs. We found that the expression of LINC81507 in tumor tissues was markedly decreased in NSCLC patients, especially in tumor tissues with lymph node metastasis (*P* < 0.01) (Fig. 1b). The same trend was observed in TNM stage (*P* < 0.05) (Fig. 1c). Table 1 showed TNM stage of all tumors. The correlated clinicopathological analysis revealed that reduced expression of LINC81507 was associated with lymph node metastasis (*P* < 0.01), distant metastasis (*P* < 0.01), and tumor invasion (*P* < 0.01). Collectively, these clinical datas suggest that the reduced expression of LINC81507 in NSCLC may be involved in NSCLC progression and metastasis. Kaplan–Meier analysis and Log-rank test revealed the correlation between LINC81507 expression and 3-year overall survival. The median survival time for the low LINC81507 expression group was shorter than the high expression group. As shown in Fig. 1d, the high expression of LINC81507 was linked to a better 3-year OS in patients with NSCLC than the low expression group (*P* < 0.05).

### LINC81507 inhibits proliferation, EMT, and metastasis in vivo

Because of reduced expression of LINC81507 in lung cancer, we further explored the role of LINC81507 in proliferative and metastatic potential in mice. First, we performed the RT-qPCR in lung cancer cell lines and normal human lung cell line HBE (Fig. 1e). A549, PC-9, and Calu-1 exhibited low expression of LINC81507 and were selected for further studies. Then, we construct the LINC81507 overexpression (LINC81507-OE) and the matching LINC81507 control (LINC81507-ctrl) cell lines for further research (additional file. 1a). Among these cell lines, the A549 variants were chosen for mouse model.

**Fig. 2:**
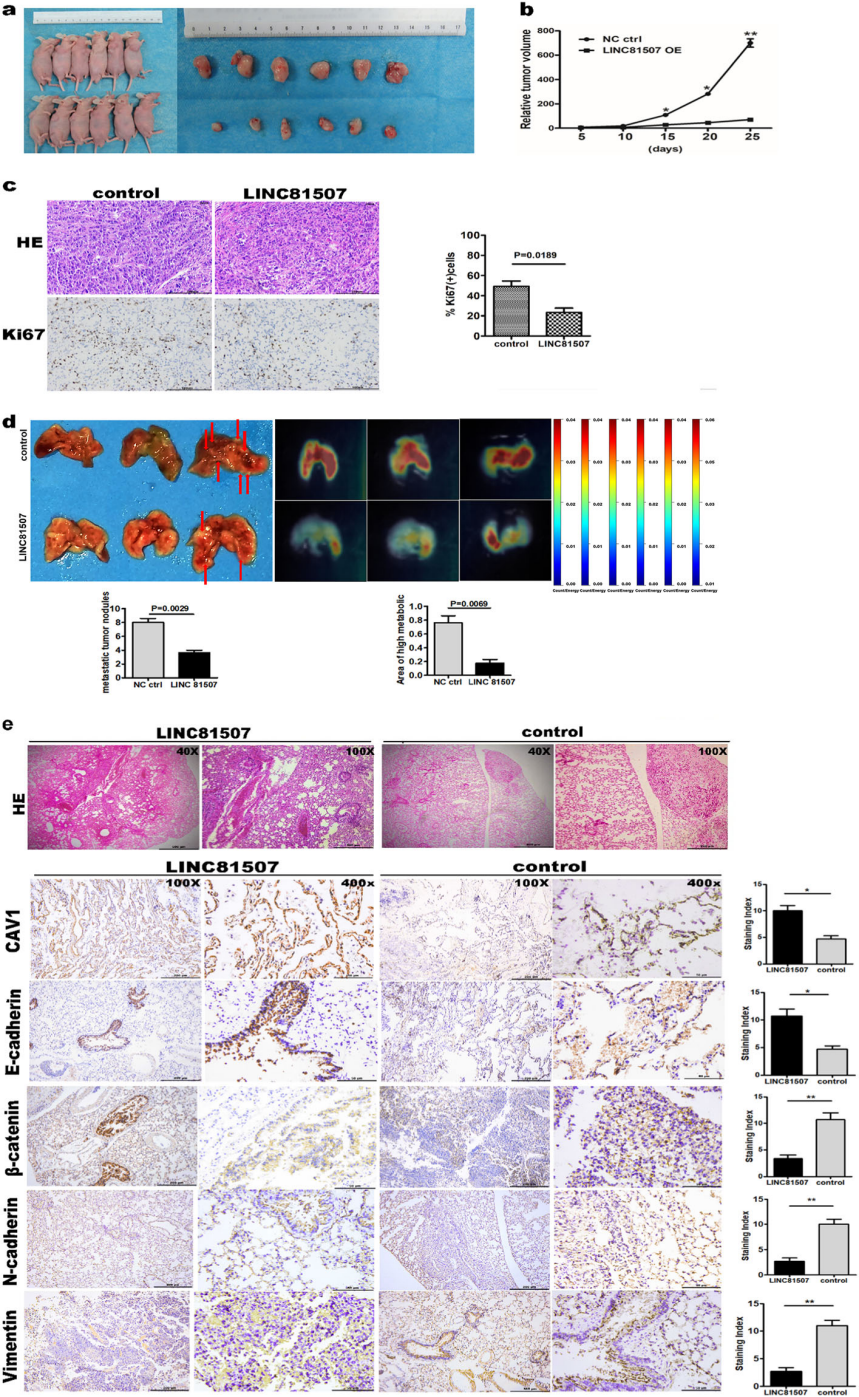
LINC81507-overexpression inhibits proliferation and metastasis in vivo. **a** Representative images of the xenograft tumors. **b** The relative volumes of tumors were analyzed. **c** Representative images of HE and immunohistochemical staining for Ki67 expression in tumor tissues, ×20 are shown. **d** LINC81507-overexpression inhibits A549 cells metastasis in vivo (*n* = 3). Representative photographs of gross lungs (left), with arrows pointing to lung surface tumor nodules, and the bioluminescent change in A549^LINC81507-OE^ group was significantly decreased compared with the vector control (right), the relative tumor nodules and high metabolic area were showed. The quantitation of lung metastasis was assessed by bioluminescence measurements. **e** H&E-stained section of lung, with original magnification: ×4, ×10 are shown. Micrograph indicates the magnified morphology of tumor tissues. Representative images of immunohistochemical staining for CAV1, E-cadherin, N-cadherin, β-catenin, and Vimentin expression in mouse metastasis lung tissues compared with normal mouse lung tissues (in ×10 and ×40). Data were represented as the mean ± SEM of three independent experiments. *P < 0.05, **P < 0.01, ***P < 0.001

As we expected, in mouse model, bioluminescence imaging showed that animals of the A549^LINC81507-ctrl^ group exhibited more lung metastatic tumors. Increased metastasis to the lung was confirmed by histological analysis (Fig. 2e). In contrast to the control group, A549^LINC81507-OE^ group showed less and smaller lung metastatic nodules (Fig. 2d). These results indicate that LINC81507 inhibited metastasis in vivo. In mouse subcutaneously tumorigenicity model, the average tumor volume of A549^LINC81507-OE^ group was obviously smaller than the tumors volume in the A549^LINC81507-ctrl^ group (*P* < 0.01) (Fig. 2a). The growth-curve of subcutaneous tumor volume was evaluated and a distinct difference was observed (Fig. 2b). The percentage of Ki-67 positive cells was decreased in A549^LINC81507-OE^ group compared with A549^LINC81507-ctrl^ group (Fig. 2c). These results provided further evidence that LINC81507 inhibits proliferation in NSCLC.

Recent evidence showed that EMT is an important process, especially in tumor metastasis^20–22^. Thus, we also examined the expression of the related EMT marker proteins in lungs by IHC assay. The expression of E-cadherin, an epithelial marker, was increased in the A549^LINC81507-OE^ tumors, while the mesenchymal marker N-cadherin, vimentin, and β-catenin were significantly decreased (Fig. 2e). These results indicate that LINC81507 inhibits proliferation and EMT in vivo.

### LINC81507 overexpression inhibits lung cancer cells’ proliferation, migration, and invasion in vitro

**Fig. 3:**
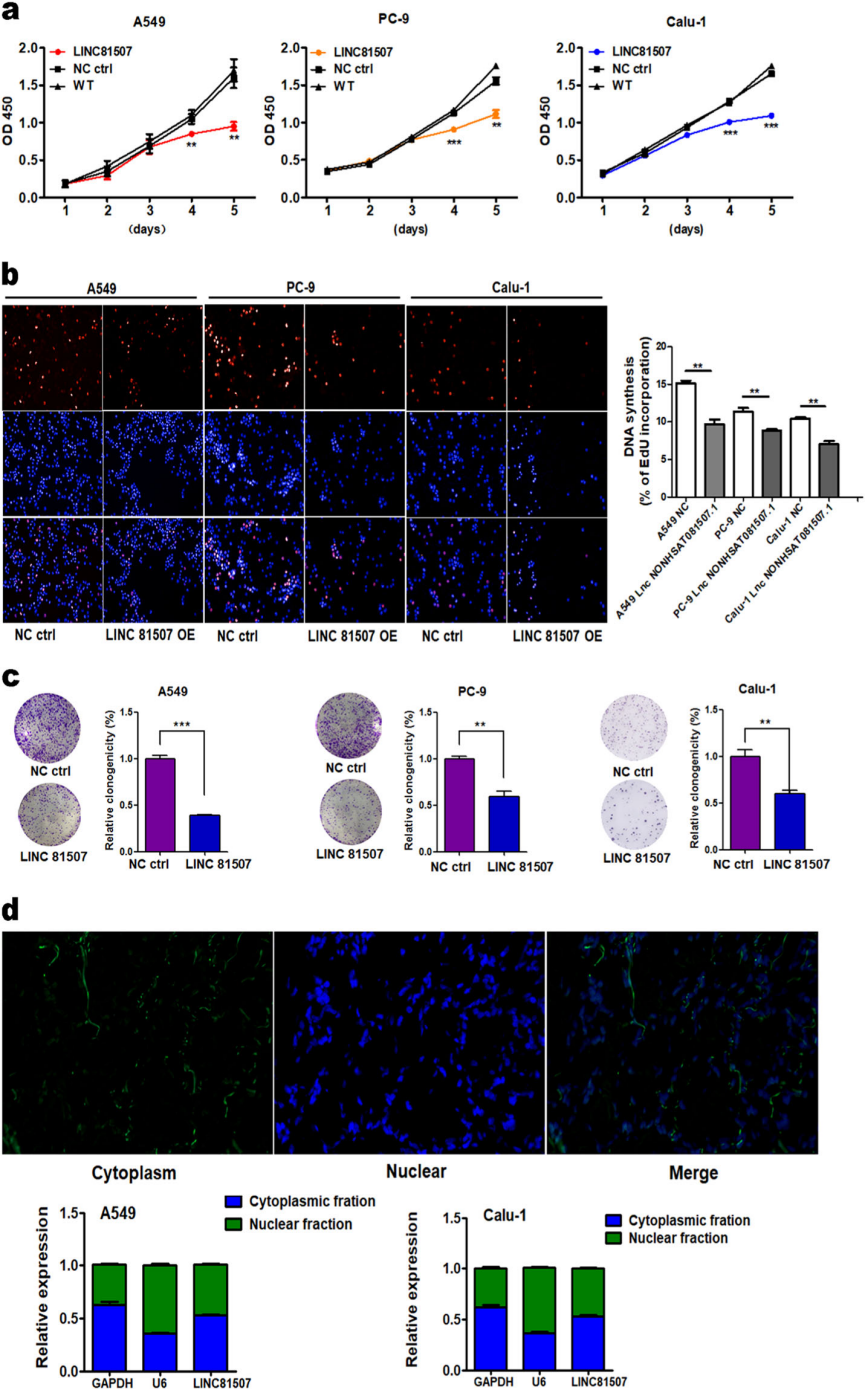
LINC81507 overexpression inhibits lung cancer cells’ proliferation and its location in tissues and cells. **a** Assessment of proliferation of A549, PC-9, and Calu-1 cells transfected with pcDNA3.1-LINC81507 or negative control by CCK-8 assay. **b** 5-ethylnyl-2ʹ-deoxyuridine (EdU) incorporation assay of A549, PC-9, and Calu-1 cells transfected with pcDNA3.1-LINC81507 or negative control. **c** Colony formation assay of A549, PC-9, and Calu-1 cells transfected with pcDNA3.1-LINC81507 or negative control. **d** Fluorescence in situ hybridization (FISH) assay of LINC81507 location in NSCLC tissues. After nuclear and cytosolic separation, RNA expression levels were measured by RT-qPCR. GAPDH was used as a cytosol marker and U6 was used as a nucleus marker. Data were represented as the mean ± SEM of three independent experiments. *P < 0.05, **P < 0.01, ***P < 0.001. LINC81507 OE means LINC NONHSAT081507.1 overexpression. WT means wild-type cell which receive no treat. NC ctrl stands for normal cell control

**Fig. 4:**
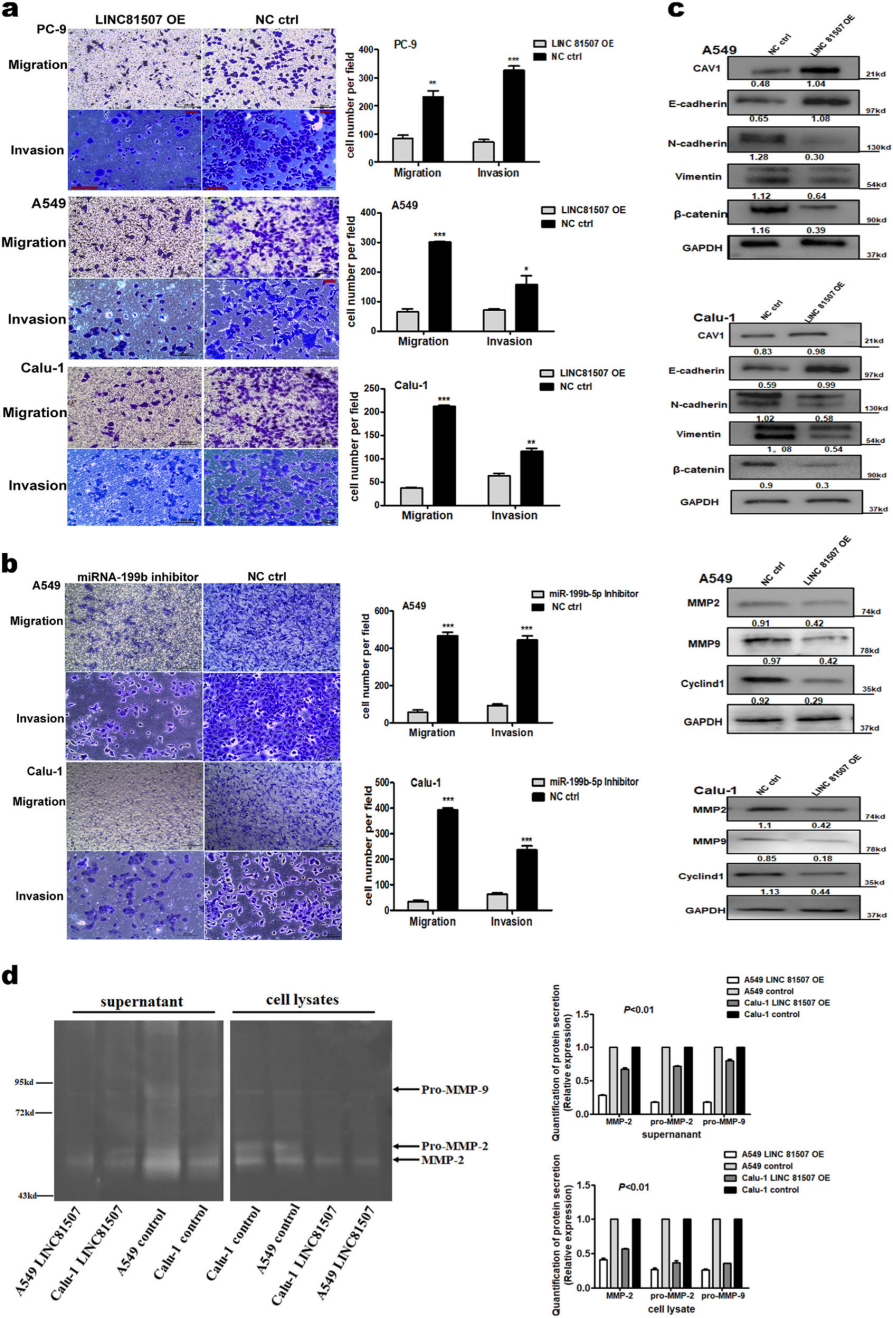
Ectopic expression of LINC81507 inhibits lung cancer cells’ migration and invasion. **a** Migration and invasion assay of A549, PC-9, and Calu-1 cells transfected with pcDNA3.1-LINC81507 or negative control. **b** Migration and invasion assay of A549 and Calu-1 cells transfected with miRNA-199b inhibitor or negative control. **c** In A549 and Calu-1 cells with LINC81507 overexpression, the protein levels of CAV1 and E-cadherin were significantly increased compared with the control, while N-cadherin, Vimentin, and β-catenin significantly decreased. Related expression of MMP2, MMP9, and Cyclin D1 protein were increased in LINC81507-overexpression group by western blot analysis. **d** Representative illustration of a zymography gel for detection of latent and active gelatinase levels ((pro-)MMP-2 and -9). The analysis was performed in culture supernatants and cell lysates of A549 and Calu-1 cells in serum-free medium for 24 h. Samples were loaded on an electrophoresis gel containing gelatin. The detected bands of MMP-2/pro-MMP-2 and the bands showed decreasing expression with LINC81507 overexpression. The same trend in Pro-MMP-9, while MMP-9 was not detectable by gelatin zymography. Data were represented as the mean ± SEM of three independent experiments. *P < 0.05, **P < 0.01, ***P < 0.001. miRNA-199b short for miRNA-199b-5p

Since the expression of LINC81507 is downregulated and correlates with NSCLC progression and metastasis, gain-of-function experiments were used to determine the role of LINC81507 in NSCLC cells’ proliferation and migration. To elucidate the relationship between LINC81507 and proliferation or metastasis of NSCLC, we performed CCK8 assay (Fig. 3a), colony formation (Fig. 3c), EdU (Fig. 3b), migration, and invasion (Fig. 4a) assays in A549, PC-9, and Calu-1 with LINC81507-OE or control. These findings indicated that overexpression of LINC81507 inhibited lung cancer cell proliferation, migration and invasion. We examined protein levels of E-cadherin, N-cadherin, vimentin, and β-catenin using western blot. Their alteration was consistent with the findings from the in vivo experiments (Fig. 4c). In addition, MMP-2, MMP-9, and Cyclin D1 that act as key players in tumor cell dissemination^23^ and proliferation^24^ in cancer were decreased in LINC81507-OE group (Fig. 4c). Moreover, overexpression of LINC81507 decreased gelatinases as shown by gelatin zymography (Fig. 4d). The lower levels of latent MMP-2 (pro-MMP-2) and latent MMP-9 (pro-MMP-9) were observed in LINC81507-OE group in both supernatants and cell lysates. Overall, these findings indicate that LINC81507 inhibited lung cancer cell’s proliferation, migration and invasion, and EMT.

### LINC81507 functions as a miR-199b-5p sponge in lung cancer cells

**Fig. 5:**
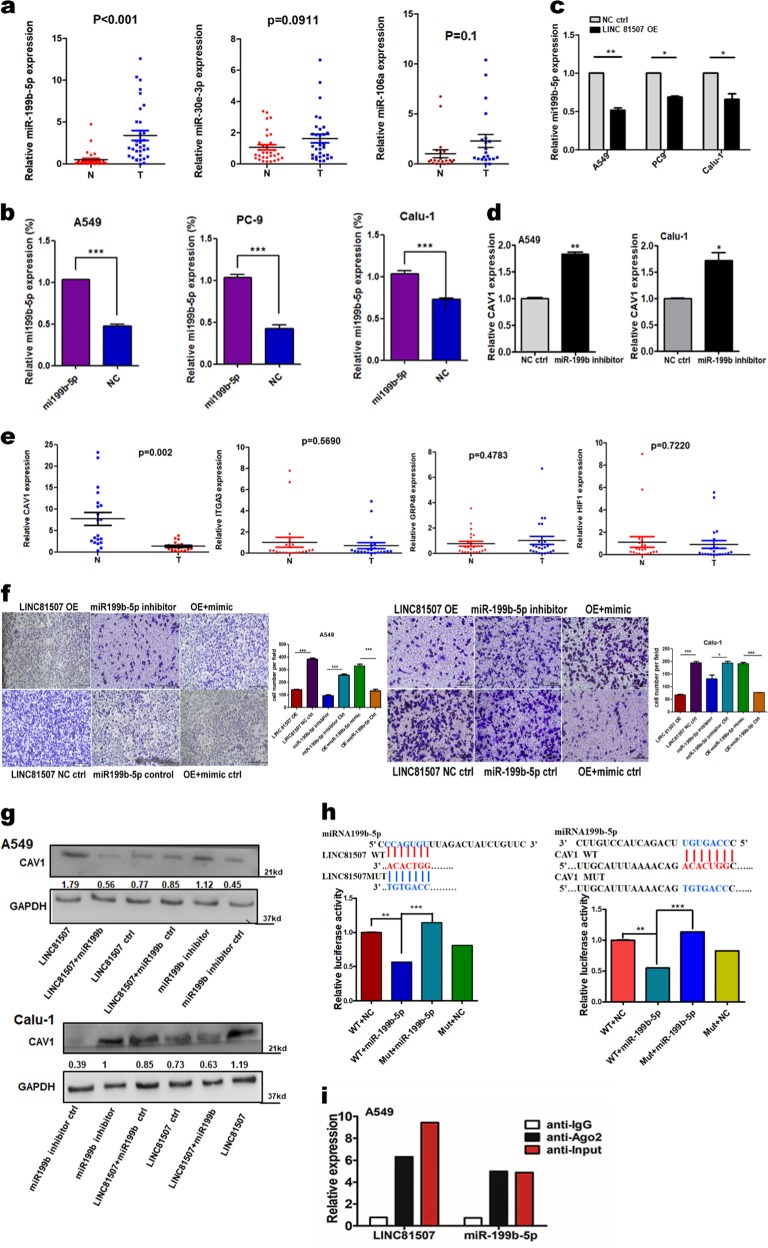
The relation between LINC81507 and miR-199b-5p. **a** Relative expression of the three indicated miRNAs from more than 20 NSCLC tumor tissues and the matched adjacent normal tissues. **b** The expressions of miR-199b-5p were analyzed using RT-qPCR in NSCLC cells. **c** The expressions of miR-199b-5p were analyzed using RT-qPCR in NSCLC cells transfected with pcDNA3.1-LINC81507 or negative control. **d** The expressions of CAV1 in NSCLC cells after decreasing miR-199b-5p. **e** The expressions of indicated mRNAs from more than 20 NSCLC. **f** Migration assay of A549 and Calu-1 cells co-transfected with pcDNA3.1-LINC81507, negative control, miRNA-199b inhibitor or miRNA-199b-5p mimic. **g** Related expression of CAV1 protein in A549 and Calu-1 cells after co-transfected with pcDNA3.1-LINC81507, negative control, miRNA-199b inhibitor or miRNA-199b-5p mimic was determined by western blot analysis. **h** Schematic of LINC81507 wild-type (WT)/mutant (Mut) and pGL3-CAV1 3′-UTR Mut/WT luciferase reporter vectors are illustrated, and the relative luciferase activities were analyzed in A549 cells co-transfected with miR-199b-5p mimics or miR-NC and luciferase reporter vectors pcDNA3.1-LINC81507-WT or Mut. **i** The RIP assay revealed that LINC81507 and miR-199b-5p were enriched in the same Ago2-immunoprecipitate. Data were represented as the mean ± SEM of three independent experiments. *P < 0.05, **P < 0.01, ***P < 0.001

Using RACE (additional file 2a), we obtained full sequence of LINC81507. Furthermore, we performed FISH assay in tissues and cytoplasmic/nuclear fractionations and showed that LINC81507 was mainly located in cytoplasm (Fig. 3d). Lots of research showed LncRNAs could function as ceRNAs to regulate target gene expression by binding to miRNAs^13^. Using bioinformatics database miRbase and TANRIC^25^ that was based on correlation coefficient, we identified top three negatively correlated miRNAs (miR-199b-5p, miR-30e and miR-106a) (additional file 6). Among these miRNAs, we further verified that miR-199b-5p expression was significantly increased in NSCLC tissues and NSCLC cells (Fig. 5a, b). We noticed an inverse correlation between miR-199b-5p and LINC81507 in transfected A549, PC-9 and Calu-1 cells (Fig. 5c). Luciferase reporter assay was used to examine the effects of LINC81507 on miR-199b-5p-regulated gene expression. The binding sites of miR-199b-5p on LINC81507 are indicated in Fig. 5h. The miR-199b-5p predicted binding site of LINC81507 (LINC81507-WT) and a mutated miR-199b-5p binding site of LINC81507 (LINC81507-MUT) were cloned into a reporter plasmid. Then, we carried out dual-luciferase assays to determine whether LINC81507 altered the reporter controlled by miR-199b-5p. Co-transfection of miR-199b-5p mimic and LINC81507-WT decreased the luciferase activity, whereas co-transfection of LINC81507-WT and miR-ctrl and co-transfection of miR-199b-5p mimic and LINC81507-MUT did not change the luciferase activity (Fig. 5h). Taken together, our data suggested a reciprocal interaction between LINC81507 and miR-199b-5p. RIP assay showed that LINC81507 and miR-199b-5p were co-immunoprecipitated through binding to Ago2 simultaneously, but not the IgG (Fig. 5i). Consistently, in migration assays, miR-199b-5p exhibited pro-migratory activity (Fig. 4b). Moreover, inhibition of migration by LINC81507 was reversed by co-transfection of miR-199b-5p mimic (Fig. 5f). Taken together, these findings suggest that LINC81507 function as a miR-199b-5p sponge in lung cancer cells.

### LINC81507 suppresses NSCLC migration and metastasis by interfering miR-199b-5p/CAV1 axis

**Fig. 6:**
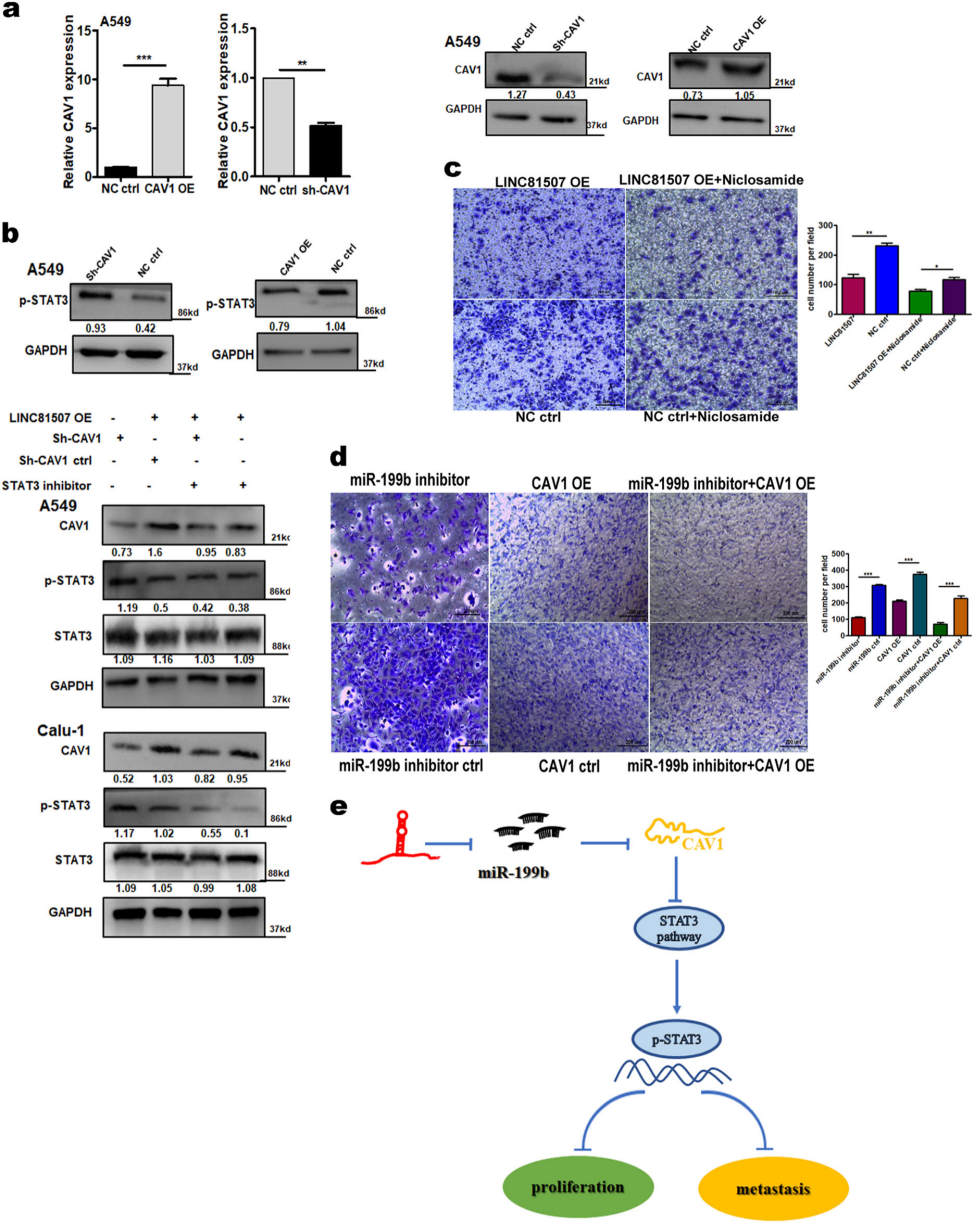
In NSCLC, LINC81507/miR-199b-5p/CAV1 axis and its STAT3 pathway. **a** The expressions of CAV1 were determined with RT-qPCR and western blot in A549 and Calu-1 cells transfected with negative control, CAV1-overexpression, or sh-CAV1. **b** Western blot assay to testify the relation between LINC81507 and STAT3 pathway. **c** Representative images and quantification of cell migration in NSCLC cells after restrain the expression of STAT3 pathway. **d** Representative images and quantification of cell migration in NSCLC cells after transfection miR-199b-5p inhibitor and/or CAV1 overexpression. **e** The schematic diagram shows the mechanism underlying LINC81507 as a ceRNA for miR-199b-5p

MicroRNAs exert their diverse biological functions mainly by degrading mRNA or suppressing mRNA translation^26^. To identify the signaling pathways that are involved in LINC81507/miR-199b-5p regulation of NSCLC proliferation and metastasis, integrated bioinformatical analysis (miRbase, Targetscan and TANRIC) were performed by our team. We identified four potential miR-199b-5p target mRNAs, Caveolin1(CAV1), ITGA3, GRP48, and HIF1. We performed RT-qPCR to examine these four mRNAs expression in NSCLC tissues. Among these mRNAs, we further verified that CAV1 expression was significantly decreased in NSCLC tissues (Fig. 5e). These results suggest miR-199b-5p has the potential to bind and reduce the expression of CAV1. Thus, we performed luciferase reporter assay (Fig. 5h) and rescue experiments (Fig. 6d) to find the relation between miR-199b-5p and CAV1. The 3′-UTR region of CAV1 mRNA, including the predicted miR-199b-5p recognition site (CAV1-WT) or the mutated sequence (CAV1-MUT), were cloned into luciferase reporter plasmids (Fig. 5h). Luciferase activity was lower in the wild-type vector than that in the mutant vector. In addition, the RNA levels of CAV1 were decreased by miR-199b-5p (Fig. 5d). Furthermore, our data proved that CAV1 is a potential target of miR-199b-5p. Then we questioned whether the CAV1 gene is regulated by the aberrant expression of LINC81507. Overexpression of LINC81507 raised the expression of CAV1 (Fig. 4c), which was consistent with the in vivo results (Fig. 2e). Consistently, miR-199b-5p inhibitor have similar effects as LINC81507 (Fig. 5g). Similarly, inhibition of migration by CAV1 could be reversed by co-transfection of miR-199b-5p inhibitor and CAV1 ctrl (Fig. 6d). These data support our speculation that LINC81507 inhibits NSCLC metastasis through the miR-199b-5p/CAV1 axis.

### LINC81507/miR-199b-5p/CAV1 axis negatively regulates metastasis via STAT3 signaling

As LINC81507/miR-199b-5p/CAV1 axis can regulate NSCLC proliferation and metastasis, we further explored the underlying signaling pathways. Research has reported that CAV1 regulates lung cancer’s apoptosis via STAT3 pathway^27–29^, which suggests that CAV1 mediate STAT3 pathway in NSCLC. Thus, we examined this axis in our study. First, we overexpressed and knockdown CAV1 (Fig. 6a). As expected, overexpression of CAV1 reduced phosphorylation of STAT3 in NSCLC (Fig. 6b), whereas reduced expression of CAV1 increased p-STAT3. These results suggest that CAV1 inhibits the STAT3 pathway in NSCLC.

Then, we hypothesized that LINC81507 can increase CAV1 via sequestering miR-199b-5p, and thereby inhibiting the STAT3 pathway and EMT in lung cancer cells. The results showed that when LINC81507 was overexpressed, the expression of p-STAT3^Y705^ was remarkably decreased (Fig. 6b). When we transfected LINC81507 with or without Niclosamide (the inhibitor of STAT3, 0.1 µM)^30–32^, the expression of CAV1 in the group with Niclosamide was lower than that without Niclosamide (Fig. 6b). We also performed the rescue experiments in the LINC85107 and Niclosamide treated groups (Fig. 6c). These data implied that LINC81507 disrupts STAT3 phosphorylation via promoting CAV1 expression. Taken together, our data suggest that LINC81507/CAV1 can block the phosphorylation of STAT3 protein, and the LINC81507/miR-199b-5p/CAV1 axis regulates NSCLC cell metastasis in a STAT3-dependent manner.

## Discussion

Recently, numerous LncRNAs have been found to regulate pathological processes in NSCLC^8^. In this study, we first discovered that LINC81507 was significantly decreased in NSCLC tissues and cell lines. LINC81507 suppressed proliferation, migration, invasion and EMT in vitro and in vivo. Our results suggest that LINC81507 acts as a tumor suppressor in NSCLC.

Compelling evidences has proven that subcellular localization determines biological function of LncRNAs^33^. The nuclear LncRNAs can regulate transcription factor directly^34^, while the cytoplasmic LncRNAs can sponge miRNA or mRNA^35^. In our experiment, we found that LINC81507 mainly resides in cytoplasm, which implies it functions as ceRNA through sponging miRNAs. Our results showed that LINC81507 functions as a ceRNA through sponging miR-199b-5p, suppressing the oncogenic role of miR-199b-5p and plays a critical role in NSCLC progression.

The previous studies have shown that miR-199 has a potential in cancer therapies, because it is associated with various tumors, including prostate cancer^36^, breast cancer^37^, medulloblastoma^38^, hepatocellular carcinoma (HCC)^39^ and osteosarcoma^40^. Our data provided the compelling evidence that miR-199b-5p expression is significantly upregulated and inversely correlated with LINC81507 expression in lung cancer cells. Overexpression of miR-199b-5p abolished the anti-metastatic ability of LINC81507. In addition, our bioinformatic analyses, co-transfection and luciferase reporter assays demonstrated that LINC81507 can directly sponge and inhibit miR-199b-5p expression.

Recent research has revealed that through degrading or inhibiting mRNAs, miRNAs act as key signal transduction mediators in tumor signaling pathways, and regulate cell fate and function^41–43^. In this study, we proved that miR-199b-5p targets the CAV1 gene directly. Our analysis also showed that CAV1 gene expression was inversely correlated with the LINC81507’s target miRNA miR-199b-5p. Upon transfection of miR-199b-5p mimic into LINC81507-OE, the expression of CAV1 was increased in A549. Although four mRNAs (CAV1, ITGA3, GRP48, and HIF1) had a potential binding site for miR-199b-5p (data not shown) in bioinformatics analysis, only the expression of CAV1 was decreased, which suggested the specificity of miR-199b-5p binding to CAV1 gene in NSCLC.

CAV1 is a multifunctional scaffolding protein and is associated with cell surface caveolae. Current research on CAV1 indicates that CAV1 can regulate multiple cancer-associated biological processes that include tumorigenesis, tumor growth, cell migration and metastasis, cell death and survival, and multidrug resistance^44^. However, the function of CAV1 is still controversial. CAV1 has been reported to enhance or inhibit various processes in tumor progression, especially as a tumor suppressor in pancreatic cancer cells^45,46^ and NSCLC cells^47–51^. The controversial function of CAV1 prompted us to explore its role in our study. In our study, the expression of CAV1 was decreased in adenocarcinoma and squamous cell carcinoma, which was congruent to the expression of LINC81507. After decreasing the expression of CAV1, the inhibitory role of LINC81507 in NSCLC progression is reversed, suggesting that upregulation of CAV1 by LINC81507 contributes to the tumor suppressor role of LINC81507 in NSCLC. We first preform migration assay in miR-199b-5p and CAV1, which proved that CAV1 had the ability to decrease migration. With respect to membrane localization of CAV1, we used TEM^52–54^ (transmission electron microscopy) to examine the membrane integrity after overexpression of LINC81507 in A549 cells. Compared with A549 control cell lines, A549 LINC81507-OE cell lines revealed less cell membrane damage and less alteration of cell wall structure by TEM (additional file. 7e), which need further researches.

Research showed that CAV1 can mediate lung cancer cells apoptosis through STAT3 pathway. Consistently our results suggest that the LINC81507/miR-199b-5p axis can inhibit the STAT3 signaling pathway by specifically regulate CAV1.

However, the potential mechanism underlying the lower expression of LINC81507 in NSCLC is still unclear, and what transcription factors regulate LINC81507 overexpression in NSCLC is still unknown. This warrants further investigation.

## Conclusions

Collectively, this is the first report that LINC81507 is decreased in NSCLC and acts as an anti-proliferative and anti-metastatic lncRNA both in vitro and in vivo. We demonstrated that through inhibiting cell cycle progression and epithelial–mesenchymal transition (EMT), LINC81507 inhibited NSCLC cell proliferation, migration, and invasion. And it acts as a ceRNA and scaffold by sponging the oncogene miR-199b-5p, and thereby suppresses NSCLC proliferation and metastasis via CAV1/STAT3 pathway (Fig. 6e). LINC81507 not only has a critical role in NSCLC progression but also provide a novel target for NSCLC diagnosis and treatment.

## Supplementary information

additional file 8

additional file 1 legend

additional file 2 legend

additional file 6

additional file 6 legend

additional file 7

additional file 7 legend

additional file 1

additional file 2

## Data Availability

All data in our study are available upon request.
