# Supplementary figures and images for: LINC81507 act as a competing endogenous RNA of miR-199b-5p to facilitate NSCLC proliferation and metastasis via regulating the CAV1/STAT3 pathway
Source: Cell Death Dis. 2019 Jul 11;10(7):533. doi: 10.1038/s41419-019-1740-9 (PMC6624296; doi:10.1038/s41419-019-1740-9)

Fig 5g:





CAV1: GAPDH:




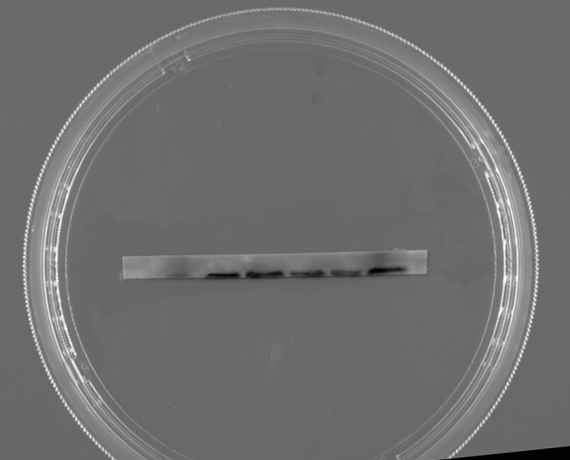
CAV1: GAPDH:

Supplement: Supplementary file 1 — additional file 8 [file 41419_2019_1740_MOESM1_ESM.docx]

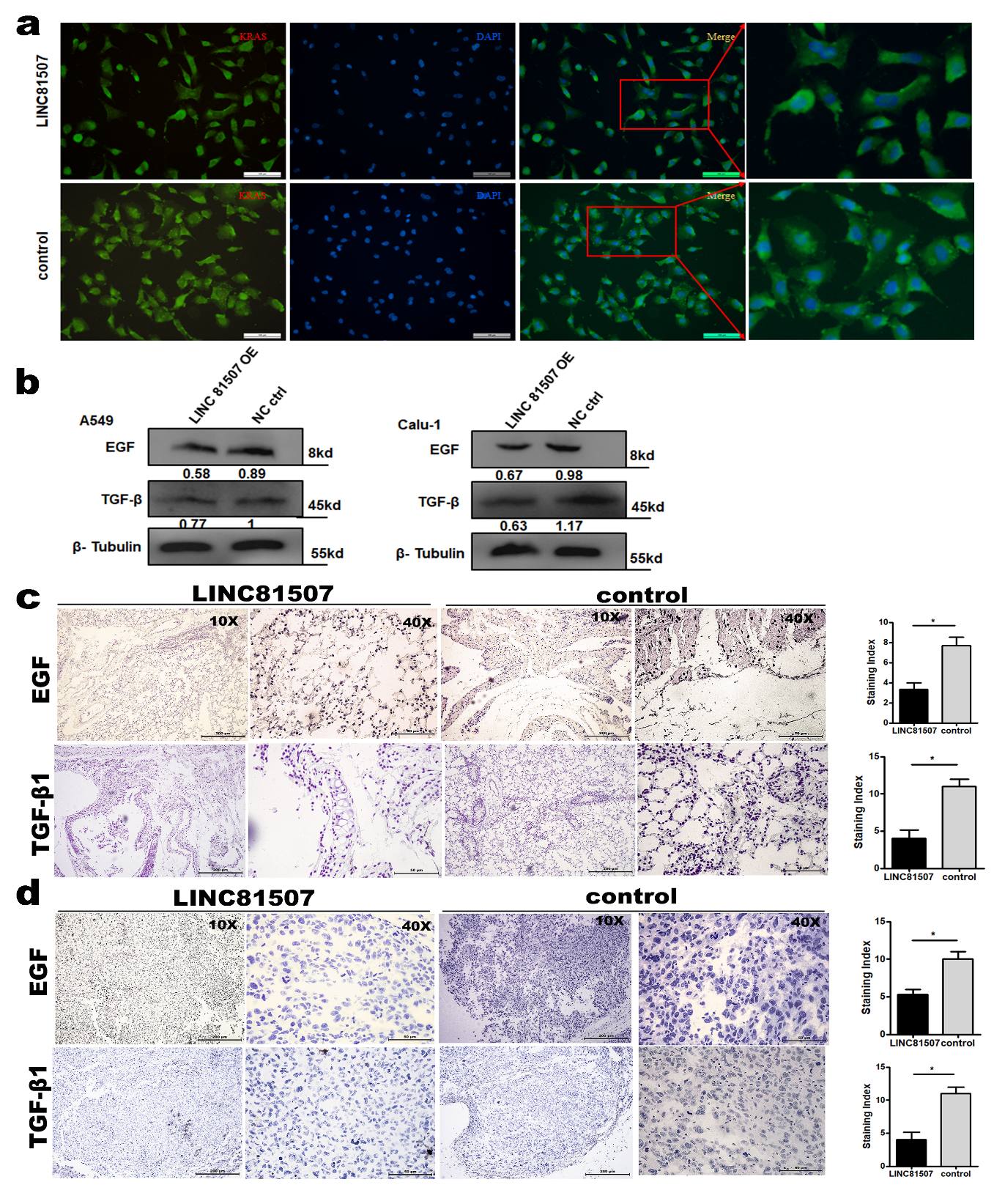

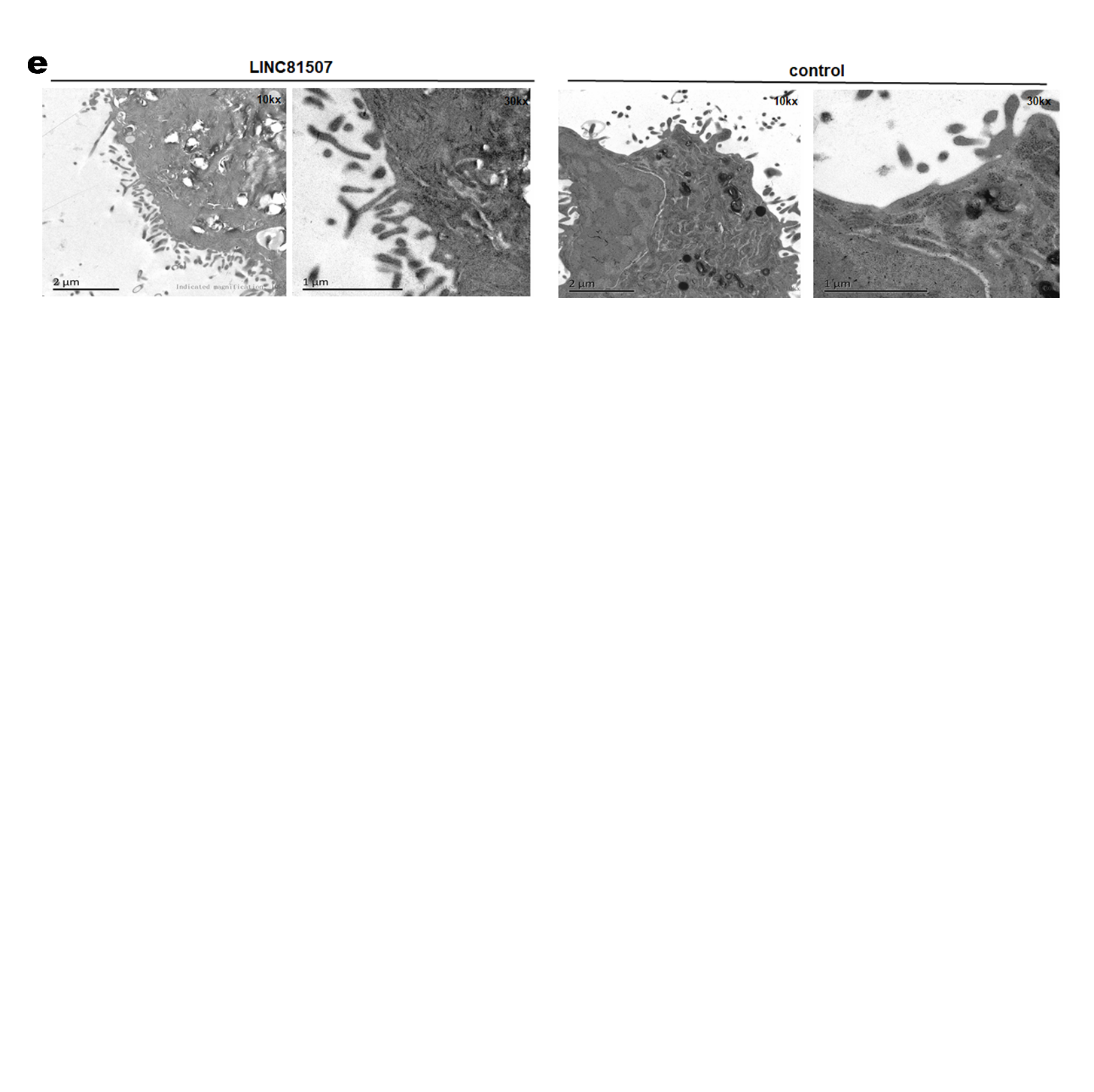

Supplement: Supplementary file 6 — additional file 7 [file 41419_2019_1740_MOESM6_ESM.docx]

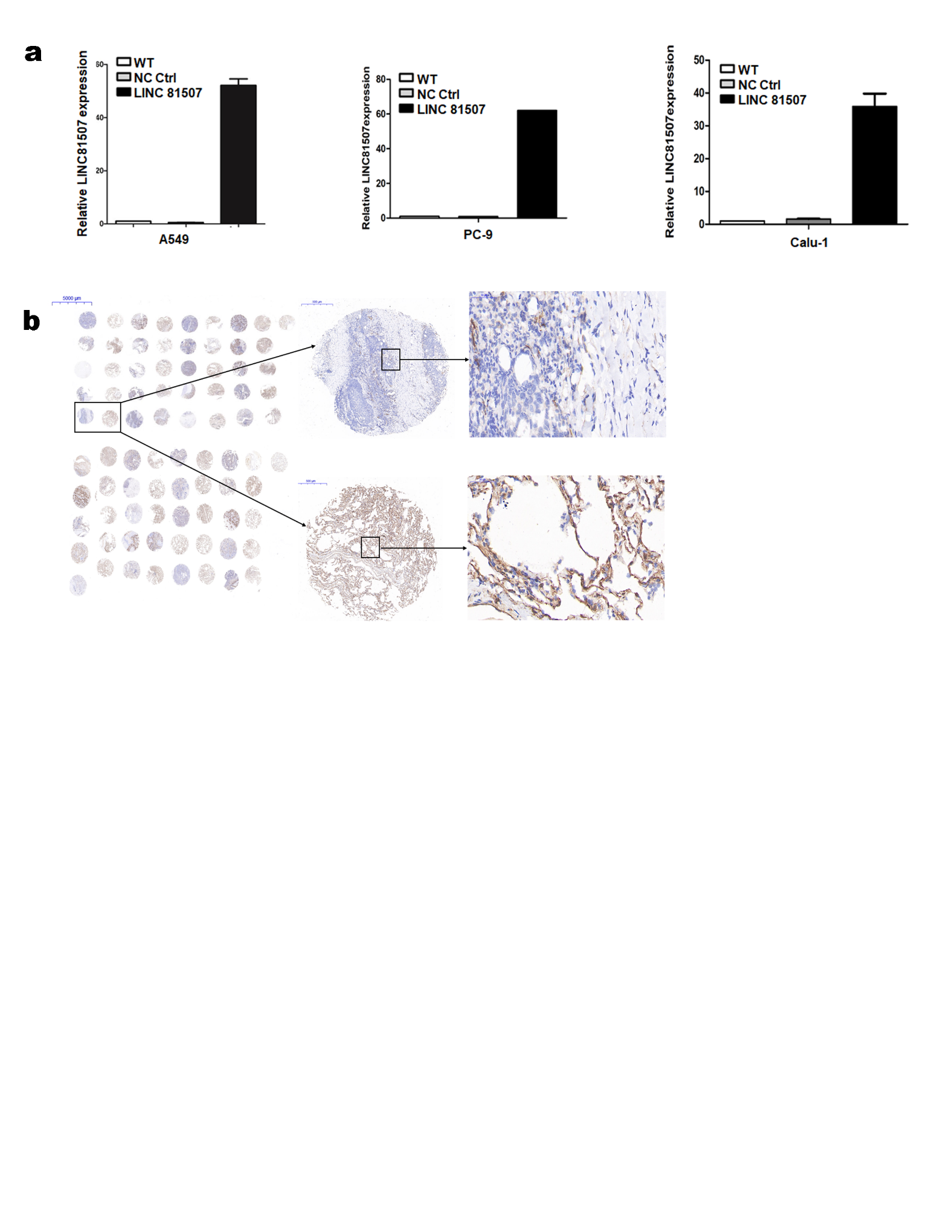

Supplement: Supplementary file 8 — additional file 1 [file 41419_2019_1740_MOESM8_ESM.docx]
