# Supplementary material for: LINC81507 act as a competing endogenous RNA of miR-199b-5p to facilitate NSCLC proliferation and metastasis via regulating the CAV1/STAT3 pathway
Source: Cell Death Dis. 2019 Jul 11;10(7):533. doi: 10.1038/s41419-019-1740-9 (PMC6624296; doi:10.1038/s41419-019-1740-9)
Supplement: Supplementary file 2 — additional file 1 legend [file 41419_2019_1740_MOESM2_ESM.docx]

Additional file 1 The expression of LINC81507 and CAV1. (a) The expressions of LINC81507 were determined with qRT-PCR in NSCLC cells transfected with pcDNA3.1- LINC81507 or negative control . (b) The immunohistochemical of CAV1 in tissue chip, which showed CAV1’s expression were decreased in lung tissues compared with ANLT.
