# Supplementary material for: LINC81507 act as a competing endogenous RNA of miR-199b-5p to facilitate NSCLC proliferation and metastasis via regulating the CAV1/STAT3 pathway
Source: Cell Death Dis. 2019 Jul 11;10(7):533. doi: 10.1038/s41419-019-1740-9 (PMC6624296; doi:10.1038/s41419-019-1740-9)
Supplement: Supplementary file 3 — additional file 2 legend [file 41419_2019_1740_MOESM3_ESM.docx]

Additional file 2 The related information about LINC81507. (a) The overall sequence of LINC81507. (b)The information of Lentiviral vector. (c) Electropherogram of enzyme digestion result. (d) The primer of LINC81507.
