# Supplementary material for: LINC81507 act as a competing endogenous RNA of miR-199b-5p to facilitate NSCLC proliferation and metastasis via regulating the CAV1/STAT3 pathway
Source: Cell Death Dis. 2019 Jul 11;10(7):533. doi: 10.1038/s41419-019-1740-9 (PMC6624296; doi:10.1038/s41419-019-1740-9)

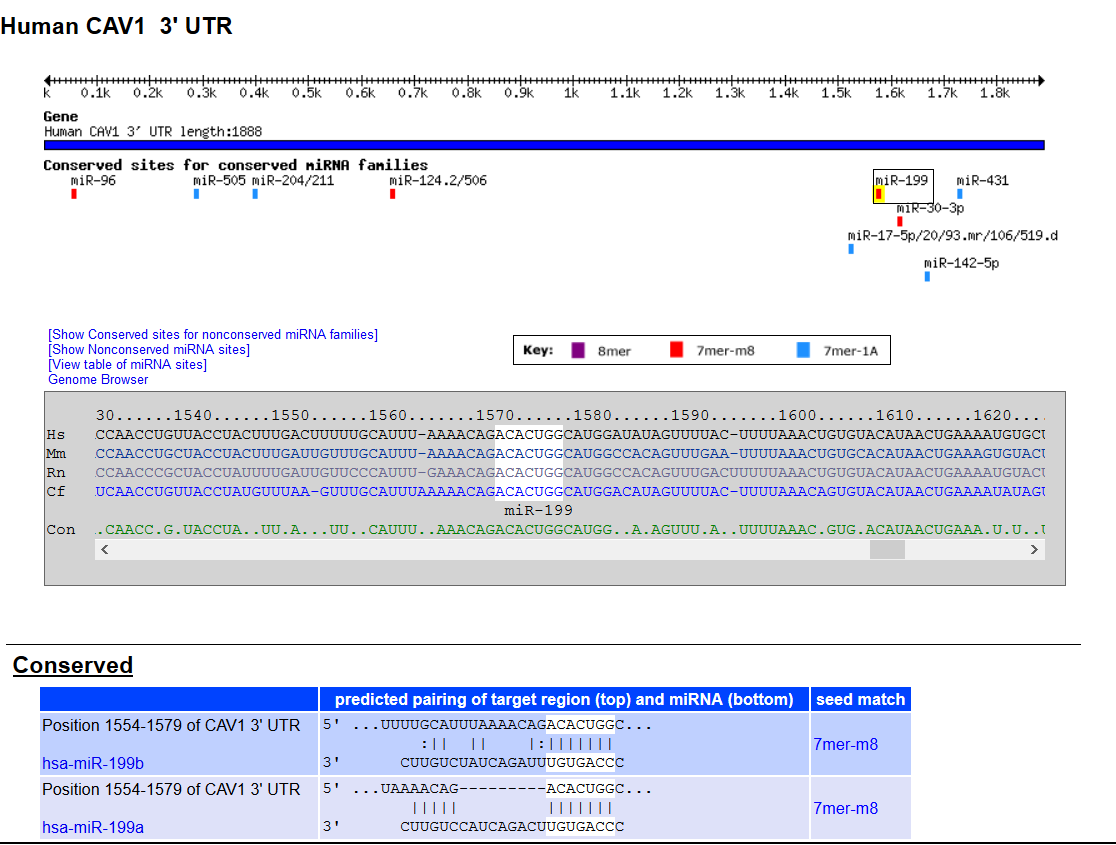


**b**

**a**

>Human NONHSAT081507.1 length: 946

TACTGTTTCCAAACTGGACACTGGAGAATATTCCTGTGAAGCCCGCAATTCTGTTGGATATCGCAGGTGTCCTGGGAAACGAATGCAAGTAGATGATCTCAACATAAGTGGCATCATAGCAGCCGTAGTAGTTGTGGCCTTAGTGATTTCCGTTTGTGGCCTTGGTGTATGCTATGCTCAGAGGAAAGGCTACTTTTCAAAAGAAACCTCCTTCCAGTAAGTATACTTTCCTACAATGCATGTCTTTCTCCTATGTCAACTAATTTTCTATTAATCATCTGTTTAAAGTCAAAAGAGAGAAGTTGGGTTATTTGTGTAGGTTTACTCTCACAAGAAGTTACTCAAGTTTATCAAGAAATACATCTGTATTAGGCTGTTCTTGCATTGCTGTAAGAAAATACCCGAGACTGGGTAGTTTATTGTAAAAAGAGGTTTAATTGGCTCACGGTTCTGCAAGCTGTACAATCCTGGCACCAACACTGCTTGGCTTTTGGGGAGGCCTCAGGAAGCTTTTTACTCATGGCTGAAGGCAAAGCGGGAGCAGGCACGTCACATGGCAAAAGCAGGAGCAAGGTTGGGGAGGTGCCACATACTTTTAAACAACCAGATTTCAAGAAAACTCAGTCACTATTCTGAGGACAGCATCAAGGGGGCGGTACTAAACCATTCATGAGAAATCCGCCCCCATGATTTAATCGGCTCCAACAAACCCCACCTCCAACATTGGGGATTACAATTCAACATAAGATTTGGGCAAGGCAAATATCCAGACTCTATCAACATCCCACTTCACACTTCATACCTACTTGTAAATGGTAGTACAGAGAGAATGATGATTACCCAGCCTACTTGTAAATGGTAGTACAGAGAGAATGATGATTACCCAGACAAGAAATATGGTCACTAATTTGATTTCATAGGCATTTGTATAAACTAACTTAAAAAA

>hsa-miR-199b-5p MIMAT0000263

CCCAGUGUUUAGACUAUCUGUUC


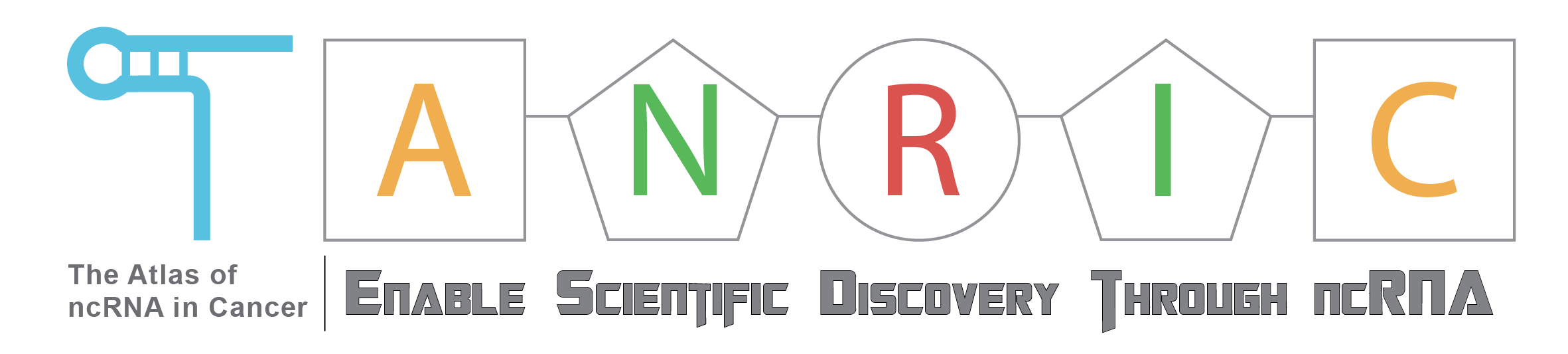

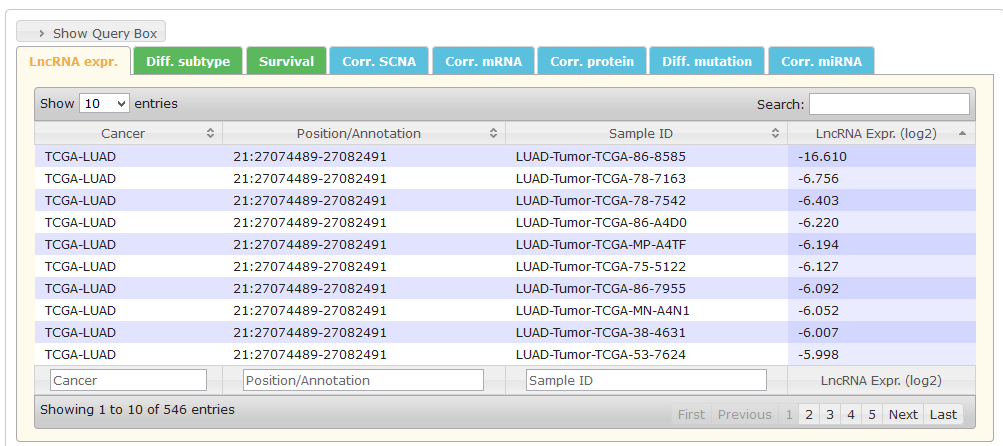

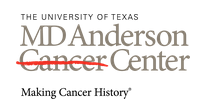


**c**


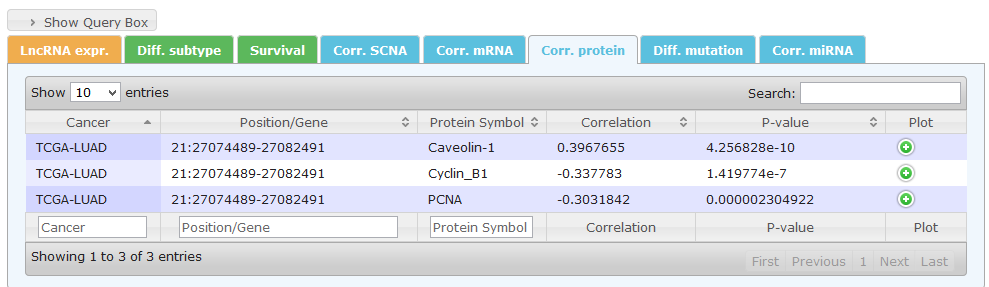


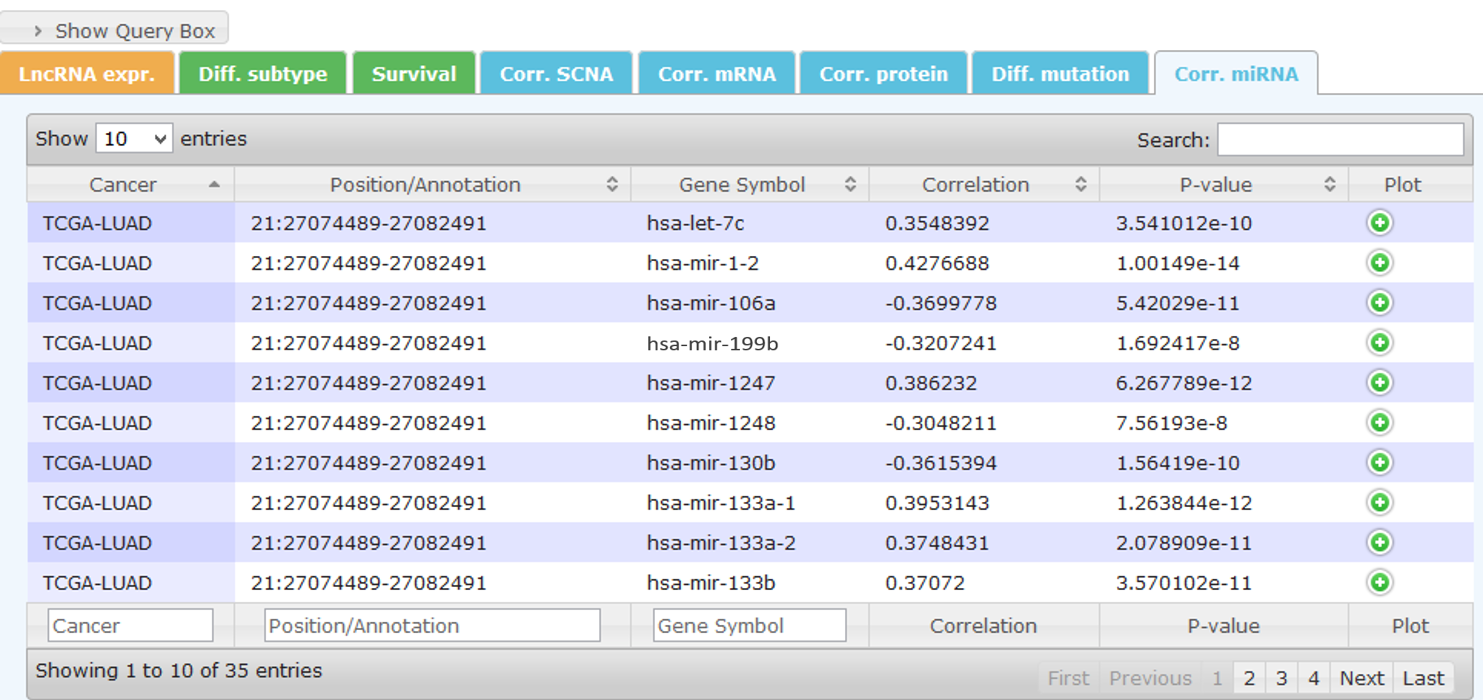

Supplement: Supplementary file 4 — additional file 6 [file 41419_2019_1740_MOESM4_ESM.docx]
