# Supplementary material for: LINC81507 act as a competing endogenous RNA of miR-199b-5p to facilitate NSCLC proliferation and metastasis via regulating the CAV1/STAT3 pathway
Source: Cell Death Dis. 2019 Jul 11;10(7):533. doi: 10.1038/s41419-019-1740-9 (PMC6624296; doi:10.1038/s41419-019-1740-9)
Supplement: Supplementary file 5 — additional file 6 legend [file 41419_2019_1740_MOESM5_ESM.docx]

Additional file 6 Bioinformatics between LINC81507, miRNA-199b-5p and CAV1. (a) The relative binding site between miR-199b-5p and CAV1. (b) Predicted binding sites between LINC81507 and miR-199b-5p (yellow color). (c) The information loaded from TANRIC database.
