# Supplementary material for: LINC81507 act as a competing endogenous RNA of miR-199b-5p to facilitate NSCLC proliferation and metastasis via regulating the CAV1/STAT3 pathway
Source: Cell Death Dis. 2019 Jul 11;10(7):533. doi: 10.1038/s41419-019-1740-9 (PMC6624296; doi:10.1038/s41419-019-1740-9)
Supplement: Supplementary file 7 — additional file 7 legend [file 41419_2019_1740_MOESM7_ESM.docx]

Additional file 7 The relation between LINC81507 and KRAS/ EGF/TGF-β1. (a) Immunofluorescence assay demonstrates that KRAS cell membrane location is markedly decreased in LINC81507-overexpression A549 cells compared with control cells. KRAS protein is stained in green color. Nuclei were counterstained with DAPI (blue). (b) Ectopic expression of LINC81507 inhibits the expression of EGF and TGF-β1 in vitro. (c) Representative images of immunohistochemical staining for EGF and TGF-β1 expression in mouse metastasis lung tissues compared with normal mouse lung tissues (in x10 and x40). (d) Representative images of immunohistochemical staining for EGF and TGF-β1 expression in tumor tissues. 4×, 10× are shown. (e) TEM images: A549 cells with LINC81507 overexpression (the left) and control cells (the right), which revealed the cell membrane damage and the change of cell wall structure in A549 control cells. 10k×, 30k× are shown.

Data were represented as the mean± SEM of three independent experiments. *P< 0.05, **P<0.01, ***P<0.001.
