# Supplementary material for: LINC81507 act as a competing endogenous RNA of miR-199b-5p to facilitate NSCLC proliferation and metastasis via regulating the CAV1/STAT3 pathway
Source: Cell Death Dis. 2019 Jul 11;10(7):533. doi: 10.1038/s41419-019-1740-9 (PMC6624296; doi:10.1038/s41419-019-1740-9)
Supplement: Supplementary file 9 — additional file 2 [file 41419_2019_1740_MOESM9_ESM.docx]

**a**

NONHSAT081507

TACTGTTTCCAAACTGGACACTGGAGAATATTCCTGTGAAGCCCGCAATTCTGTTGGATATCGCAGGTGTCCTGGGAAACGAATGCAAGTAGATGATCTCAACATAAGTGGCATCATAGCAGCCGTAGTAGTTGTGGCCTTAGTGATTTCCGTTTGTGGCCTTGGTGTATGCTATGCTCAGAGGAAAGGCTACTTTTCAAAAGAAACCTCCTTCCAGTAAGTATACTTTCCTACAATGCATGTCTTTCTCCTATGTCAACTAATTTTCTATTAATCATCTGTTTAAAGTCAAAAGAGAGAAGTTGGGTTATTTGTGTAGGTTTACTCTCACAAGAAGTTACTCAAGTTTATCAAGAAATACATCTGTATTAGGCTGTTCTTGCATTGCTGTAAGAAAATACCCGAGACTGGGTAGTTTATTGTAAAAAGAGGTTTAATTGGCTCACGGTTCTGCAAGCTGTACAATCCTGGCACCAACACTGCTTGGCTTTTGGGGAGGCCTCAGGAAGCTTTTTACTCATGGCTGAAGGCAAAGCGGGAGCAGGCACGTCACATGGCAAAAGCAGGAGCAAGGTTGGGGAGGTGCCACATACTTTTAAACAACCAGATTTCAAGAAAACTCAGTCACTATTCTGAGGACAGCATCAAGGGGGCGGTACTAAACCATTCATGAGAAATCCGCCCCCATGATTTAATCGGCTCCAACAAACCCCACCTCCAACATTGGGGATTACAATTCAACATAAGATTTGGGCAAGGCAAATATCCAGACTCTATCAACATCCCACTTCACACTTCATACCTACTTGTAAATGGTAGTACAGAGAGAATGATGATTACCCAGCCTACTTGTAAATGGTAGTACAGAGAGAATGATGATTACCCAGACAAGAAATATGGTCACTAATTTGATTTCATAGGCATTTGTATAAACTAACTTAAAAAA

**
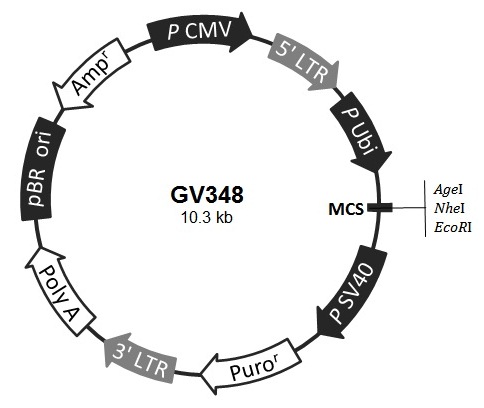
**

**b**

**c**

**
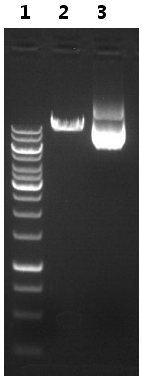
**

1#: 10kb Marker （10kb、8kb、6kb、5kb、4kb、3.5kb、3kb、2.5kb、2kb、1.5kb、1kb、750bp、500bp、250bp)

2#: Vector digestion product

3#: Untreated digestion vector

**d**

**Primer**

| **ID** | **seq** |
| --- | --- |
| lncRNA(25845-1)-P1 | CCAACTTTGTGCCAACCGGTTACTGTTTCCAAACTGGACACTGG |
| lncRNA(25845-1)-P2 | CACACATTCCACAGGAATTCTTTTTTAAGTTAGTTTATACAAATGCC |
